# Lonely and scrolling during the COVID-19 pandemic: understanding the problematic social media use and mental health link among university students

**DOI:** 10.3389/fpsyt.2024.1247807

**Published:** 2024-01-31

**Authors:** Leen K. Ghanayem, Holly Shannon, Lida Khodr, Robyn J. McQuaid, Kim G.C. Hellemans

**Affiliations:** ^1^ Department of Neuroscience, Carleton University, Ottawa, ON, Canada; ^2^ University of Ottawa Institute of Mental Health Research, Ottawa, ON, Canada; ^3^ School of Psychology, University of Ottawa, Ottawa, ON, Canada

**Keywords:** coping, problematic social media use, COVID-19, university students, stress, loneliness

## Abstract

**Introduction:**

Undergraduate university students experienced many academic and non-academic stressors during the first year of the coronavirus (COVID-19) pandemic, putting them at a greater risk of negative mental health outcomes. Reports worldwide have shown high incidences of depressive, anxiety, and stress scores among university students at the beginning of the pandemic. Emerging evidence also suggests that to cope with the stress and loneliness of the pandemic, many youth and young adults increased the amount of time they spent on social media platforms.

**Methods:**

Undergraduate students participated in an online study aimed to understand the link between time spent on social media, coping through the use of social media and problematic social media use (PSMU) with mental health symptoms, such as stress, depression, anxiety, and loneliness, during the COVID-19 pandemic.

**Results:**

While time spent on social media was only weakly associated with stress, depression, anxiety and loneliness scores, PSMU more strongly mapped onto these outcomes. Additionally, students who were coping highly using social media displayed elevated stress, depression, anxiety and loneliness levels in comparison to those reporting low levels of coping with social media. Finally, students who reported high levels of coping using social media displayed higher PSMU scores, with this relationship appearing more pronounced in students who had higher levels of loneliness.

**Conclusion:**

These data support evidence that it is not necessarily time spent on social media but rather PSMU that is relevant for mental health symptoms, and that PSMU is exacerbated by loneliness. Moreover, the current results highlight the effects of maladaptive coping on mental health symptoms and PSMU among university students during the COVID-19 pandemic.

## 1 Introduction

University students face a variety of new challenges, which can include leaving their families and support systems, financial stressors, and adapting to a faster pace of learning and academic pressures (1, 2). This transition period marks a critical period of increased vulnerability to mental disorders, with data from university student populations suggesting that 1 in 3 students struggle with a mental health or substance use disorder (3). In 2022, 32.0% and 24.6% of Canadian post-secondary students reported having been either diagnosed with anxiety or depressive disorders, respectively (4). Further, more than half of those students screened positive for loneliness, with rates appearing similar across males and females (4). Positive associations between loneliness, stress, anxiety and depressive symptom scores are frequently reported in the general population (5–7). Specifically, loneliness is strongly related to depression and anxiety symptoms in young adults and mediates the relationship between social skills, including social sensitivity, expressivity and control, and both anxiety and depression disorders (8, 9). Although the association between loneliness and both depression and anxiety symptoms are strong, these associations may differentially relate to mental health symptoms. For example, loneliness mediated the relationship between social components and depressive symptoms, but not anxiety symptoms (10). Similarly, the relationship between loneliness and depression symptoms, but not anxiety, was significantly stronger in females (8). Nevertheless, the relationships between loneliness and mental health symptoms appear strong and bidirectional (11).

In an effort to deal with reduced social networks and loneliness owing to moving away from families and friends in this transitional period, many university students use social media as a coping method (12). However, social media is viewed as a maladaptive coping method to relieve negative emotions, such as rumination and worry (13–15). In fact, high emotional and social stress are predictive of social media use behavior in adolescents and young adults (16). Moreover, social media use can predict mental health symptoms, such as anxiety and depression, and using social media significantly increases stress (17). Smartphone use is also associated with higher depression and anxiety symptoms and lower quality of sleep (18).

Increasingly, scholars are recognizing a pattern of use associated with social media that resembles a substance use disorder (19). In this regard, problematic social media use is characterized by preoccupation, excessive use that affects mood states, and withdrawal symptoms (20). Delineating between screen time, frequency of use or PSMU is important in terms of the contributions to mental health (21). For example, associations between depression and anxiety and use of multiple social media platforms remain strong even after controlling for time spent on social media (22). Using multiple social media platforms is a possible indicator of intensity of use as it has been frequently associated with negative mental health outcomes (23, 24). In addition, time spent on social media only weakly correlates with mental health symptoms, whereas problematic use may represent a more detrimental pattern of social media use (25, 26). To further support this claim, recent meta-analyses suggest that PSMU has a moderate association with depression, anxiety, stress and loneliness symptoms, whereas time spent on social media only weakly relates to depression, loneliness and psychological well-being (21, 27, 28).

In a social media-driven era, it is important to understand the effects that these platforms have on mental health outcomes, particularly during stressful experiences and among populations at risk, such as young adults. The link between loneliness and social media use is evident (29, 30), whereby perceived loneliness predicted excessive social media use and anxiety, with excessive social media use further increasing anxiety levels (28). Further, loneliness is not only associated with both social media use and psychological distress but also mediated the relationship between passive social media use and distress (31). Emerging evidence also suggests that as a means to cope with the stress and loneliness of the COVID-19 pandemic, many youth and young adults increased the amount of time they spent on social media platforms (8, 32, 33). In addition, loneliness during the pandemic was associated with increasing mental health symptoms, where students reporting anxiety or depressive symptoms were six to eight times more likely to report loneliness (34, 35). Consequently, many youth and young adults increased the amount of time they spent on social media platforms as a means to cope with the stress and loneliness of the COVID-19 pandemic (28, 36, 37). Exposure to social media during previous epidemics, such as the Ebola outbreak, and terrorist attacks, such as the Boston Marathon bombings in 2013, are strongly tied to increased anxiety and psychological distress (38, 39). More recent reports during the COVID-19 pandemic revealed associations between perceived stress and PSMU (40). Furthermore, data exploring mental health and loneliness during the pandemic among young adults show high levels of loneliness and stress, particularly among individuals aged 18-29 years old (8, 36), which was also associated with the use of social media as a method of coping (34, 36). Additionally, individuals who reported higher PSMU during the pandemic also reported higher psychological distress (41).

The current literature suggests a relationship between the experiences of the pandemic and increased mental health symptoms among university students and particularly young women (8, 42). Further, social media exposure during the pandemic among students has, in fact, coincided with higher anxiety and depressive scores (43). However, very few studies to this date explore the relation between coping strategies used by university students and mental health symptoms during the pandemic. Current evidence also suggests an impact of coping and stressors, including loneliness, on different substance disorders, such as alcohol use, gambling disorder and problematic video game use (44–46). Moreover, evidence in the literature reveals an impact of coping with social media and stress symptoms on PSMU (40). However, there remains a lack of evidence explaining the impact of coping with social media and loneliness on PSMU. Since recent reports have indicated that students have increased their time spent using social media (36), the current study focuses on exploring the relationships between using social media as a coping strategy during the pandemic and mental health symptoms. Due to the clear relationship between stress, loneliness, and social media use, it was of interest to explore whether high levels of these mental health symptoms during the pandemic would exacerbate the relationship between using social media as a coping method and PSMU. We predicted that students who report using social media as a coping method would report greater stress, mental health symptoms, and loneliness scores. In addition, we predicted that stress and loneliness symptoms would moderate the relationship between coping by using social media and PSMU.

## 2 Methods

### 2.1 Participants and procedure

Students enrolled at Carleton University, Ottawa, Ontario participated in an online study via Carleton University’s Psychology study system (SONA). Inclusion criteria comprised any undergraduate student aged 18-29, who had not participated in any previous wave of this study. Participants completed informed consent online followed by a series of online questionnaires which included questions pertaining to PSMU, loneliness, stress, and mental health, as well as questions developed to assess the negative impacts of the COVID-19 pandemic across a number of domains (for more information see Prowse et al., 2021). This study took place from September 2020 to December 2020 during the COVID-19 pandemic. For context, this time period coincides with increased restrictions following a surge in COVID-19 cases in Canada and around the world. In Ontario, where the majority of participants resided (90.6%, *n* = 730), increased restrictions were implemented between October 2020 and November 2020, which included tighter restrictions on social gatherings between different households (47). Carleton University students were learning almost exclusively online. Students participating and completing this study received a 0.5% credit in their courses. This study was approved by the Carleton University Research Ethics Board (REB #111775).

### 2.2 Measures

#### 2.2.1 Demographics

Participants completed basic demographic questions related to sex/gender identity, age, ethnicity, year of study, and living arrangements. They were also asked questions pertaining to their general mental and physical health.

#### 2.2.2 Depressive, anxiety and stress symptoms

The Depression, Anxiety and Stress Scale, version 21 (DASS-21) (48) was used to measure states of depression, anxiety and stress using three sub-scales. The responses ranged from “low” (0) to “high” (3) to assess depressive, anxiety and stress symptoms. The stress subscale assesses chronic arousal symptoms, such as difficulty relaxing, nervous arousal, impatience, irritability, and agitation (α_Stress_ = .88). The depression subscale assesses depression symptoms, such as dysphoria, hopelessness, devaluation of life, self-deprecation, lack of interest/involvement, and anhedonia (α_Depression_ = .93). Finally, the anxiety subscale assesses anxiety symptoms, such as autonomic arousal, skeletal muscle effects, situational anxiety, and the subjective experience of anxious affect (α_Anxiety_ = .86).

#### 2.2.3 Loneliness

The 20-item UCLA Loneliness Scale (UCLAL) (49) was used to measure loneliness. Answers were recorded on a range of “Never” (1) to “Always” (4). Higher scores correspond to a higher incidence of loneliness (α = .91).

#### 2.2.4 Problematic social media use

The Bergen Facebook Addiction Scale (BFAS) (50) was used to assess PSMU. The 18-item scale was modified by replacing “Facebook” with “social media.” The answers were recorded on a scale ranging from “Very rarely” (1) to “Very often” (5). The total scores for the 18-item scale were calculated and reported in the results section. However, based on the authors’ recommendation, 6 items were used to calculate problematic use: items 1, 5, 7, 11, 13 and 16. These items reflect each of the 6 core elements of addiction: salience, mood, modification, tolerance, withdrawal, conflict, and relapse. Students who had a score of 3 or more on at least 4 out of 6 questions were considered to meet the criteria of PSMU (50). Higher scores indicated a higher incidence of PSMU (α = .79).

#### 2.2.5 Social media use

To assess social media use patterns, participants were asked: “How many hours a day do you currently spend on social media altogether (Facebook, Instagram, Snapchat, Twitter, etc.)?” “What social media platform do you spend the most time using?” and “What is currently the main reason you use social media?” In addition, to understand how social media use patterns changed since the pandemic, participants were asked: “Has this [hours spent on social media] increased since the COVID-19 pandemic?” and “If yes, how many extra hours has this increased by?”

#### 2.2.6 COVID-19 pandemic questionnaire

Questions assessing the impacts of the COVID-19 pandemic were used to examine how the pandemic had impacted different aspects of participants’ daily lives across a number of domains. The answers to the questions include five options ranging from “not at all” (0) to “an extreme amount” (4). Furthermore, questions assessing coping with the COVID-19 pandemic were also included. This scale was used previously in a study assessing gender differences in student mental health and coping during a different wave of the pandemic (36). For the purpose of this study, only questions related to coping by using social media were included in the analyses. Responses were collapsed into three categories for analyses: “not at all/a little” (0), “a moderate amount” (1), and “very much/an extreme amount” (2). However, responses were collapsed into two categories for moderation analyses specifically: “not at all/a little/a moderate amount” (1) and “very much/an extreme amount” (2).

### 2.3 Statistical analysis

All statistical analyses were performed using SPSS for Mac OS 27.0 (SPSS Science, Chicago, IL, USA). For data cleaning, all items were checked for out-of-range scores. Moreover, all outliers (± 3.29) were brought into range. To assess relations between measures, correlational analyses were performed using Pearson correlation coefficients. Analyses examining whether differences in coping by social media use related to stress, depression, anxiety and loneliness scores were conducted using univariate Analysis of Variance (ANOVA). Significant main effects were followed up using Bonferroni *post-hoc* tests. While we recognize that gender identity occurs on a spectrum, due to a small number of participants that did not gender identify as male or female, (*n* = 13), they were not included in statistical analyses that specifically examined gender differences as a grouping factor, however, were included in all other analyses. Moderation analyses were conducted using model one in PROCESS (51) to examine the moderating role of loneliness and stress symptoms in the relation between coping by using social media and PSMU. Statistical significance was determined at *p* <.05 (two-tailed).

## 3 Results

### 3.1 Participant demographics

**Table 1** describes the characteristics of participants in this study. Participants (*N* = 806) in the current study were undergraduate students aged 17-29 (*M* = 19.14, *SD* = 2.18). Of participants, 72.2% identified as female (*n* = 582), 26.2% as male (*n* = 211), 0.6% as non-binary (*n* = 5), 0.4% as gender-fluid (*n* = 3), 0.2% as transgender (*n* = 2), 0.1% as gender-nonconforming (*n* =1), and 0.2% identified their gender as other (*n* = 2). Participants came from diverse ethnic backgrounds with 61.3% identifying as White (*n* = 494), 7.3% as Asian (*n* = 59), 7.2% as Arab (*n* = 58), 6.6% as Black (*n* = 53), 5.8% as South Asian (*n* = 47), 2.7% as South East Asian (*n* = 22), 2.1% as Latin American (*n* = 17), 2.0% as Indigenous (*n* = 16) and 5.0% of participants reported their ethnicity as other, which largely comprised mixed ethnicity (*n* = 40). The majority of participants in this study lived with their parents (69.7%, *n* = 562), whereas others reported living with roommates off-campus (13.2%, *n* = 106), alone in residence (5.8%, *n* = 47), with roommates in residence (3.5%, *n* = 28), with a spouse/significant other (3.0%, *n* = 24), alone off-campus (2.9%, *n* = 23), and with children either alone or with a spouse/significant other (0.1%, *n* = 1/each).

**Table 1:** Demographic characteristics of university students in this study.

Characteristics	Mean	SD
Age, years	19.14	2.18

	Frequency (*n*)	Percentage (%)
Gender		
Female	582	72.2
Male	211	26.2
Non-binary	5	0.6
Gender-fluid	3	0.4
Transgender	2	0.3
Gender non-conforming	1	0.1
Other	2	0.2
Ethnicity		
White	494	61.3
Asian	59	7.3
Arab	58	7.2
Black	53	6.6
South Asian	47	5.8
South East Asian	22	2.7
Latin American	17	2.1
Indigenous	16	2.0
Other	40	5.0
Living Condition		
With parents	562	69.7
With roommates off-campus	106	13.2
Alone in residence	47	5.8
With roommates in residence	28	3.5
With spouse/significant other	24	3.0
Alone off-campus	23	2.9
With child alone	1	0.1
With child and significant other	1	0.1

### 3.2 Social media use among university students

As shown in **Table 2**, participants reported varying levels of social media use per day, with the most common response being three or four hours a day, (range = 0 to more than 8 hours/day). Of participants who reported using social media, 77.5% reported that their use had increased since the beginning of the pandemic (*n* = 625), with the most common response being that their use increased by one to two hours/day. When asked about the social media platform students spent the most time using, the top reported social media application was Instagram followed by Snapchat, TikTok, Facebook, YouTube, and Twitter. Finally, when asked about listing the main reasons for using social media, the most common responses included keeping in touch with friends and family (37.5%, *n* = 302), fun/entertainment (26.0%, *n* = 210), and boredom/distraction (23.1%, *n* = 186).

**Table 2:** Characteristics of social media use patterns among university students, including time spent on social media and most commonly used social media applications.

Social Media Use	Frequency (*n*)	Percentage (%)
Hours a day spent on social media altogether (Facebook, Instagram, Snapchat, Twitter, etc.)?		
No social media use	19	2.4
One	50	6.2
Two	130	16.1
Three	165	20.5
Four	151	18.7
Five	118	14.6
Six	84	10.4
Seven	31	3.8
Eight or more	58	7.2
If your time spent on social media per day increased since the pandemic, how many extra hours has this increased by?		
One to two hours	389	48.2
Two to three	130	16.1
Three to four	49	6.0
More than four hours	34	4.2
Which social media platform do you spend the most time using?		
Instagram	300	37.2
Snapchat	242	30.0
TikTok	241	29.9
Facebook	45	5.5
YouTube	43	5.3
Twitter	34	4.2
What is currently the main reason you use social media?		
Keep in touch with friends and family	302	37.5
Fun/Entertainment	210	26.0
Boredom/Distraction	186	23.1
Other	108	13.40

The average problematic social media raw score in this sample was 14.72 ± 5.75. Males and females significantly differed, *t* (777) = -7.54, *p* <.001, with females (*M* = 15.60, *SE* = .23) scoring significantly higher than their male counterparts (*M* = 12.21, *SE* = .37). Of all participants, 37.75% of students (*n* = 299) had a score of 3 or higher on 4 out of the 6 questions (50).

### 3.3 Mean scores on depression, anxiety, stress and loneliness outcomes

**Table 3** shows the mean stress, anxiety, depression and loneliness scores in this sample, with symptoms ranging from mild to moderate. Particularly, the mean loneliness score in the sample was considered moderate. It was of interest to further explore gender differences in the mean stress, anxiety, depression and loneliness scores as shown in **Table 3**. Upon examining stress, anxiety and depression scores, females had significantly higher stress (*t* (515.92) = -10.88, *p* <.001), anxiety, (*t* (546.11) = -9.04, *p* <.001), depression (*t* (445.16) = -6.90, *p* <.001) and loneliness (*t* (791) = -3.58, *p* <.001) scores in comparison to their male counterparts.

**Table 3:** Mean gender differences for stress, anxiety and depression symptoms and loneliness.

Variables	*Mean*	*SE*	Female		Male		*t-test*	*p*
			*Mean*	*SE*	*Mean*	*SE*		
**Stress (DASS-Stress)**	11.97	.36	13.74	.43	6.43	.51	-10.88	<.001
**Anxiety (DASS-Anxiety)**	8.01	.32	9.28	.38	3.99	.44	-9.04	<.001
**Depression (DASS-Depression)**	12.35	.41	13.63	.48	9.60	.66	-6.90	<.001
**Loneliness (UCLAL)**	45.75	.42	46.49	.48	43.09	.85	-3.58	<.001

### 3.4 Relationship between time spent using social media, problematic social media use and mental health

Correlational analyses examining the relationship between stress, depression, loneliness, time spent on social media and PSMU scores are shown in **Table 4**. Importantly, it is apparent that PSMU related more strongly with mental health and loneliness scores than did simply assessing the time spent on social media. Specifically, PSMU mainly had moderate associations, whereas time spent using social media had very weak correlations with outcome measures.

**Table 4:** Pearson correlation coefficients between stress, depression, loneliness, and PSMU.

	1	2	3	4	5	6
**1.Stress (DASS)**	–					
**2. Depression (DASS)**	.77**	–				
**3. Anxiety (DASS)**	.80**	.70**	–			
**4. Loneliness (UCLAL)**	.47**	.61**	.43*	–		
**5. Time spent using social media**	.11*	.11**	.07**	.09**	–	
**6. Problematic Social Media Use (BFAS)**	.31**	.31**	.27**	.26**	.28**	–

**p <.001, *p <.01.

Hierarchical regressions were also conducted to explore time spent using social media and PSMU as unique predictors of mental health outcomes and loneliness symptoms, as shown in **Table 5**. The overall model was significant for stress, *R^2^_change_* = .08, *F* (2, 786) = 42.75, *p* <.001, anxiety, *R^2^_change_* = .07, *F_change_* (2, 786) = 31.57, *p* <.001, depression, *R^2^_change_* = .08, *F_change_* (2, 785) = 42.22, *p* <.001 and loneliness, *R^2^_change_* = .06, *F_change_* (2, 787) = 28.91, *p* <.001. Specifically, time spent using social media independently predicted the mental health symptoms and loneliness, as shown in step 1 in **Table 5**; however, once PSMU was added to the model in step 2, only PSMU significantly predicted stress, anxiety, depression and loneliness symptoms, while time spent using social media no longer significantly predicted outcome variables.

**Table 5:** Linear regression models of predictors of stress, anxiety, depression and loneliness symptoms.

	*b*	*SE B*	*β*	*95% CI*	*p-value*
Stress					
*Step 1*					
Constant	9.41	.85		[7.75, 11.07]	<.001
Time spent using social media	.63	.18	.12	[.27,.99]	<.001
*Step 2*					
Constant	6.88	.86		[5.18, 8.57]	<.001
Time spent using social media	.19	.18	.04	[-.16,.55]	.29
Problematic Social Media Use	1.57	.18	.30	[1.21, 1.93]	<.001
Anxiety					
*Step 1*					
Constant	6.61	.74		[5.15, 8.07]	<.001
Time spent using social media	.35	.16	.08	[.03,.66]	.03
*Step 2*					
Constant	4.61	.76		[3.11, 6.11]	<.001
Time spent using social media	.001	.16	.00	[-.32,.32]	.99
Problematic Social Media Use	1.24	.16	.27	[.92, 1.56]	<.001
Depression					
*Step 1*					
Constant	9.43	.94		[7.58, 11.29]	<.001
Time spent using social media	.71	.20	.12	[.31, 1.11]	<.001
*Step 2*					
Constant	6.68	.96		[4.78, 8.57]	<.001
Time spent using social media	.24	.20	.04	[-.16,.64]	.24
Problematic Social Media Use	1.71	.20	.29	[1.30, 2.11]	<.001
Loneliness					
*Step 1*					
Constant	43.26	.98		[41.33, 45.18]	<.001
Time spent using social media	.59	.21	.09	[.17, 1.00]	.006
*Step 2*					
Constant	40.81	1.01		[38.83, 42.80]	<.001
Time spent using social media	.16	.21	.03	[-.26,.58]	.45
Problematic Social Media Use	1.52	.22	.25	[1.09, 1.95]	<.001

### 3.5 Coping with the COVID-19 pandemic

When participants were asked about using social media to deal with the stress of the pandemic, 59.7% reported using it “very much” or “an extreme amount” (*n* = 481), 24.4% reported “a moderate amount” (*n* = 197), and 15.9% reported “a little” or “not at all” (*n* = 128). These results differed significantly by gender*, χ^2^*_(2, N = 793)_ = 38.85 = *p* <.001. As expected, females were significantly more likely to report using social media as a coping method “very much” or “an extreme amount” (65.1%, *n* = 379), compared to males (45.5%, *n* = 96).

**Figure 1:**
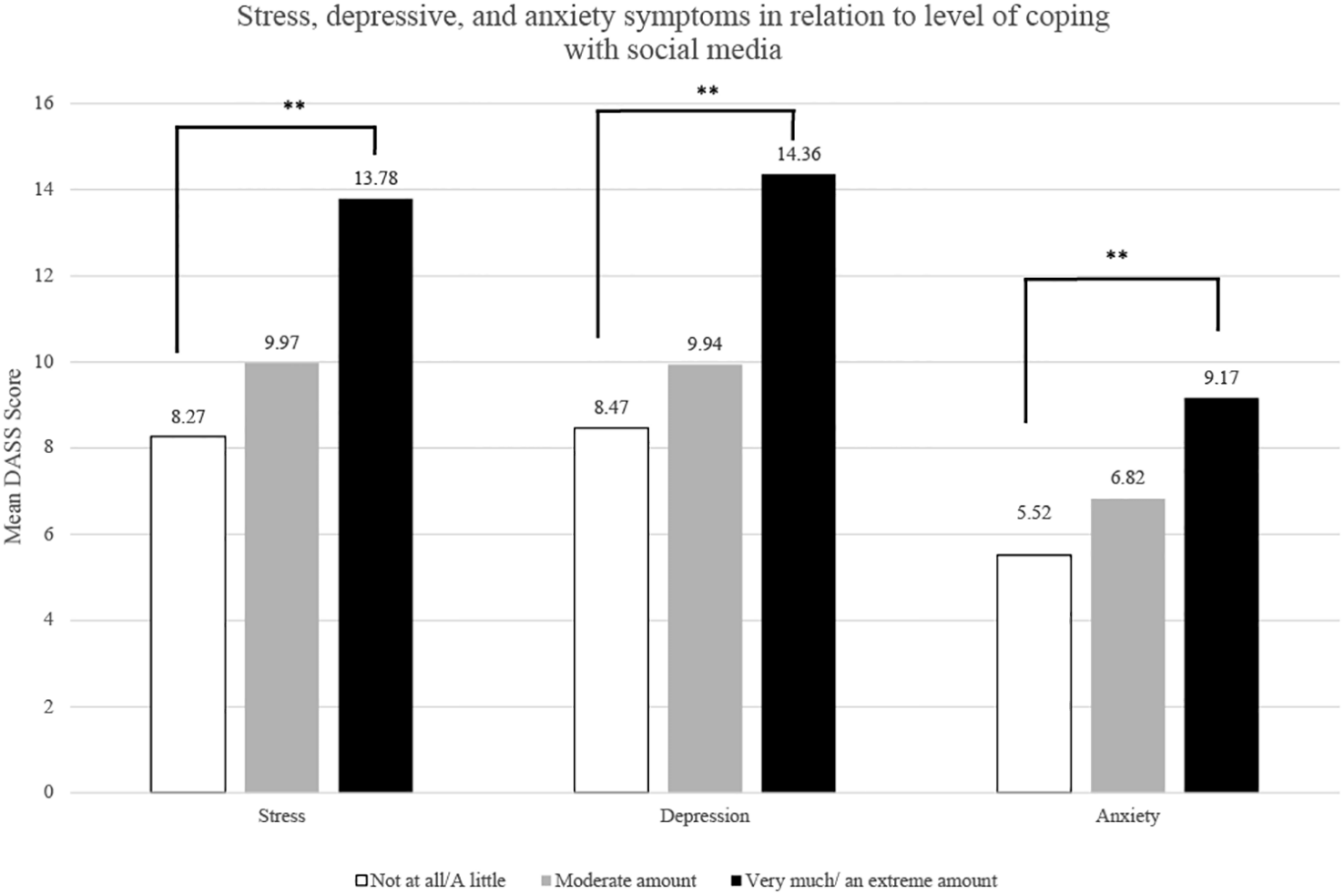
Mean stress, depression, and anxiety scores in relation to different levels of coping via social media use. **p <.001 relative to students who reported “not at all” or “a little” to coping via social media use.

**Figure 2:**
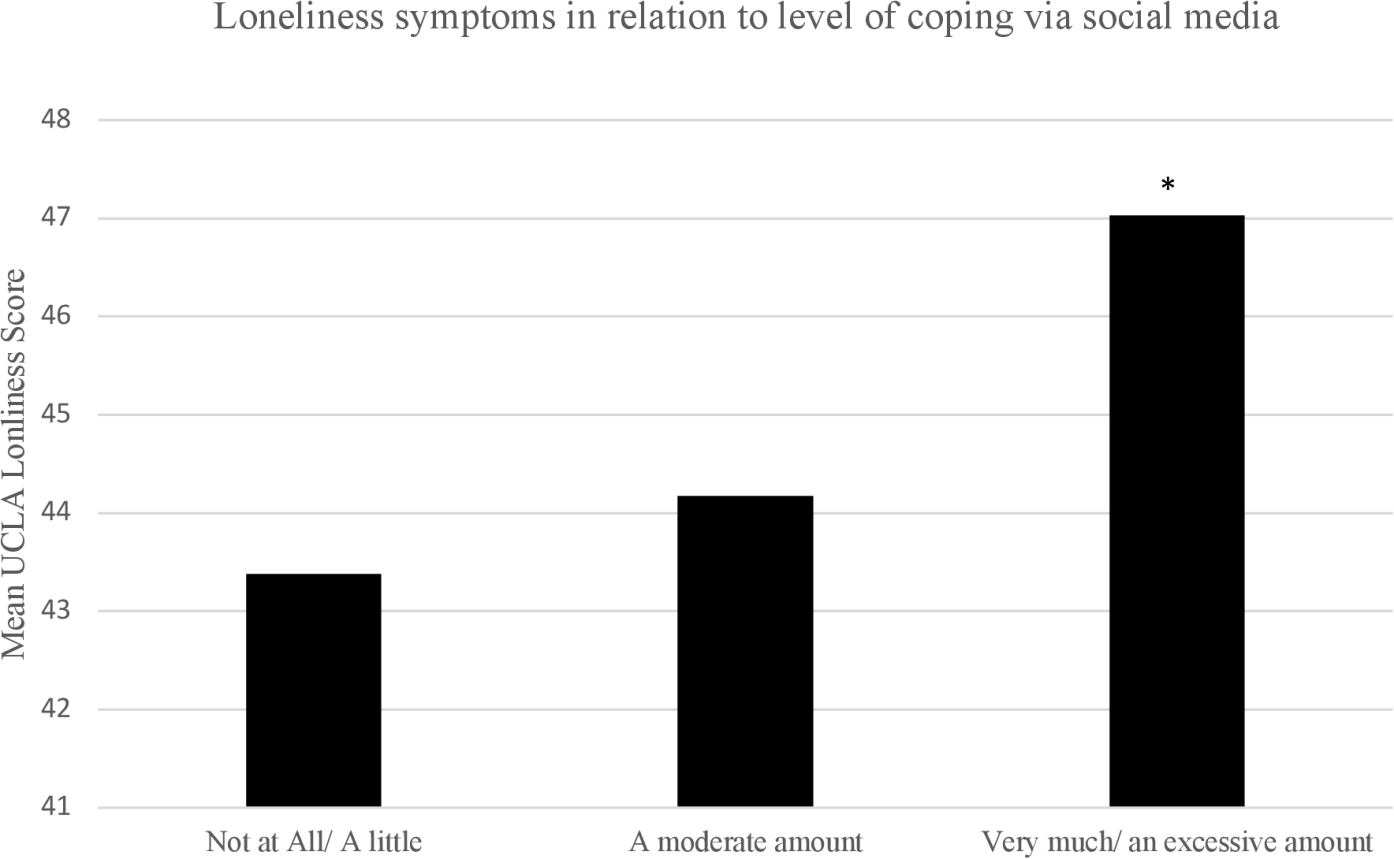
Mean loneliness scores in relation to different levels of coping via social media use. *p <.01 relative to students who reported “not at all” or “a little” to coping via social media use.

Univariate ANOVA analysis was used to examine whether the extent of coping via social media use related to stress, anxiety, depression, and loneliness symptoms. Self-reported stress, *F* (2,802) = 20.17, *p* <.001 *_partial_η^2^* = .05, anxiety, *F*(2,802) = 10.78, *p* <.001, *_partial_η^2^* = .03 and depression symptoms, *F*(2,801) = 19.66, *p* <.001, *_partial_η^2^* = .05, differed significantly depending on the extent of coping. As shown in **Figure 1**, *post-hoc* analyses revealed that students who reported using social media to cope with the pandemic “very much” or “an extreme amount” had significantly higher stress, anxiety and depression scores compared to students who reported using social media “not at all” or “a little”, all *p’s* <.001. However, stress, anxiety and depressive symptoms in students who reported using social media to cope with the pandemic “a moderate amount” did not differ significantly from those who reported using social media “not at all” or “a little”, *p* = .41, *p* = .60 and *p* = .76. respectively. As shown in **Figure 2**, loneliness scores also differed according to the extent of coping by social media use, *F* (2,803) = 7.14, *p* <.001, *_partial_η^2^* = .02. Specifically, students who reported coping via social media “very much” or “an extreme amount” had significantly higher loneliness scores (*M* = 47.03, *SE* = .54) compared to those who reported coping via social media “not at all” or “a little” (*M* = 43.38, *SE_s_* = 1.05), *p* = .006, or “a moderate amount” (*M* = 44.17, *SE*= .844), *p* = .01.

### 3.6 Coping via social media and problematic social media use

**Figure 3:**
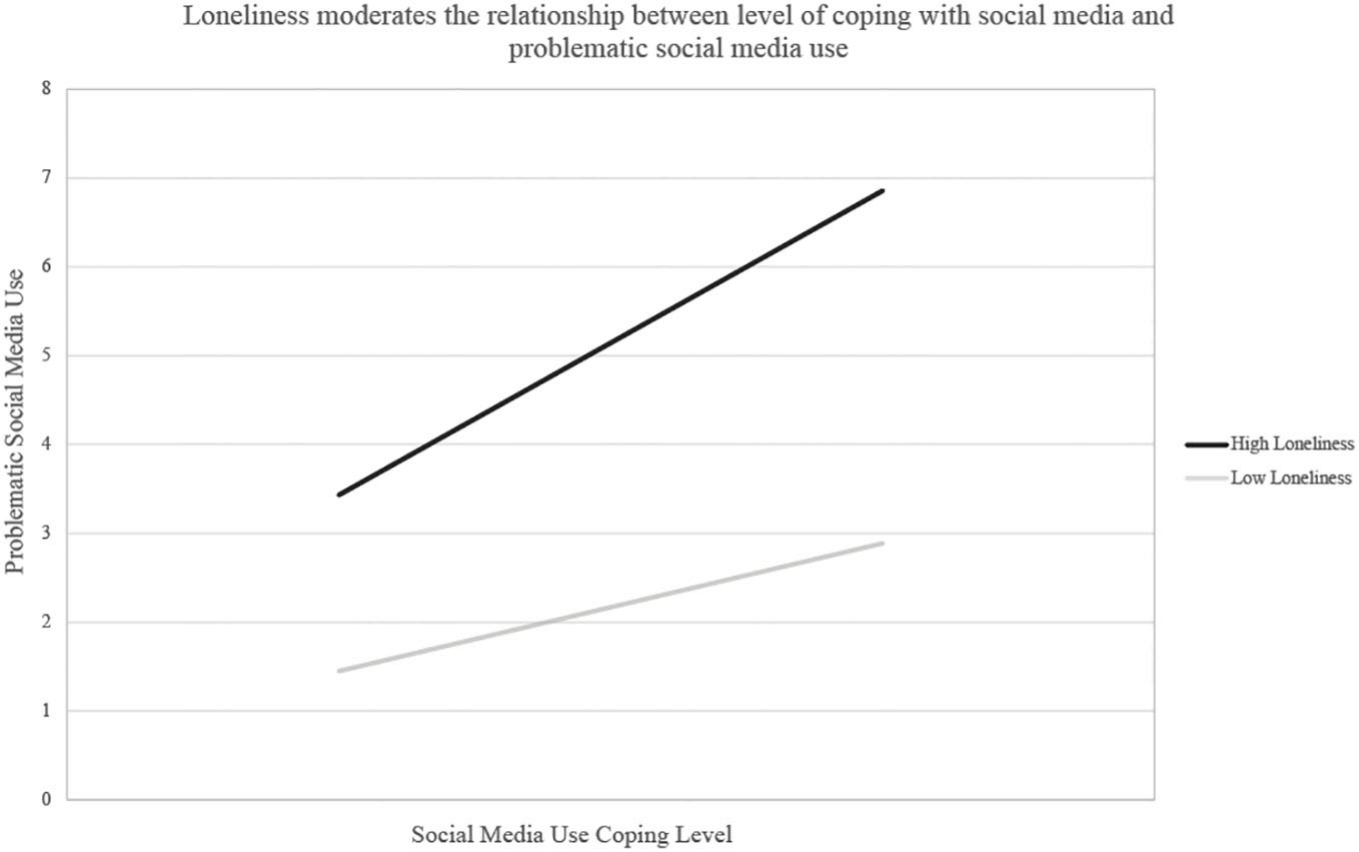
Loneliness as a moderator between coping via social media and PSMU.

The level of coping via social media during the pandemic differed significantly on PSMU scores, *t* (788) = -14.07, *p* <.001. As expected, students reporting high levels of coping with social media (*M* = 3.50 *SE* = .08) had significantly higher problematic social media scores compared to students who reported low social media coping levels (*M* = 1.68, *SE* = .10). Given that very few studies examine the role of stress and loneliness symptoms in the relationship between coping with social media and PSMU, it was of interest to examine whether these symptoms would exacerbate this relation. When examining whether stress levels moderated the relation between coping via social media and PSMU, it was determined that stress did not differentially influence this relationship, *R^2^_change_* = .001, *F* (3, 786) = 1.39, *p* = .24. However, loneliness did moderate the relationship between coping via social media and PSMU. The overall model was significant, *R^2^* = .24, *F* (3, 786) = 84.96, *p* <.001, as was the interaction, *R^2^_change_* = .004, *F* (1, 786) = 4.28, *p* = .04. Specifically, and as shown in **Figure 3**, while the relationship between coping via social media and PSMU was significant at low levels of loneliness, *B* = 1.44, [95% CI (1.09, 1.79)], *p* <.001, this relationship was stronger at high levels of loneliness, *B* = 1.98, [95% CI (1.62, 2.35)], *p* <.001.

## 4 Discussion

The current study aimed to explore the relationship between using social media as a coping strategy during the COVID-19 pandemic, problematic social media use, and mental health. In our study, the majority of students indicated their use of social media had increased since the pandemic, with 48.2% indicating their use of social media had increased by one to two hours per day since the pandemic. In addition, 39% of students had reported using social media for three to four hours per day. The most common reasons reported for using social media were “entertainment/fun”, “connecting with friends and family,” and “boredom.” In the current study, more than half of all students (59.7%) reported high rates of coping with social media, and this was more commonly reported among women. This is in line with other reports during the pandemic that show women were more likely to use social media to cope with social isolation during the pandemic in comparison to their male counterparts (36, 52). This could be explained by the fact that women are more sensitive to negative mental health outcomes due to unmet or poor social connections, despite having larger social support systems in comparison to men (53).

When we explored how and whether social media related to mental health symptoms among a population of university students, we found a moderate relation between PSMU and higher depressive and stress scores and a weak relation between PSMU and both anxiety and loneliness scores. In contrast, time spent on social media was very weakly correlated with all mental health symptoms. Further examination of the relationship between time spent using social media, PSMU and mental health symptoms revealed that time spent no longer significantly predicts these symptoms when assessed with PSMU. These results further support evidence in the literature that it is not time spent on social media but rather the pattern of use of social media that is associated more strongly to mental health symptoms (21). As PSMU describes a more clinically relevant behavior, based on the core elements of addiction, it encompasses not only time spent using social media but also other behaviors such as withdrawal and tolerance. Further, evidence from the literature around substance use disorders suggests that coping motivations associated with substance use relate more strongly to problematic use (54, 55). Increasingly, literature exploring social media use seems to suggest that coping motivations (i.e., using to cope with symptoms of ill mental health) may also relate to problematic use (40). Restrictions associated with the COVID-19 pandemic created an environment wherein many endorsed social media as a form of coping (37). The lack of social and peer connections among university students in particular was associated with increased mental health challenges (36, 56). In our study, students who reported using social media more to cope with the pandemic had significantly higher depressive, anxiety, stress and loneliness symptoms, although effect sizes were small, accounting for only 2-5% in the outcomes assessed. Notably, the severity of the mental health outcomes in students differed according to their coping levels. While students reporting low to moderate coping with social media showed normal levels of stress, anxiety, and depressive symptoms, students who reported high levels of coping showed mild to moderate symptom severity. These results support that excessive usage of social media to cope is a form of maladaptive coping as it relates to an increase in mental health symptoms.

Additionally, students who indicated that they used social media to cope with the stress of the pandemic also had significantly higher PSMU scores compared to students who reported low usage of social media to cope. Since the pandemic, there has been a global rise in PSMU rates, in tandem with an increase in stress, anxiety and depressive symptoms as a result of lockdown measures (57, 58). Evidence suggests the relationship between using social media and mental health outcomes may be bidirectional, where some individuals use social media to cope with negative mental health outcomes, such as stress or depressive symptoms, while others experience increasing negative mental health symptoms as a result of excessive social media use. Using social media as a coping method to relieve negative mental health outcomes may also worsen these symptoms, further increasing social media use (40). These behaviors mirror the positive and negative reinforcement processes described in the formation of substance use disorders (59). During the pandemic, individuals who were highly stressed and used social media for entertainment or social motives (i.e., positive reinforcement) or to cope with feelings of isolation or perceived stress (i.e., negative reinforcement) showed significantly higher PSMU scores (40). These and our data support and extend previous research demonstrating that coping motives associated with substance use (e.g., alcohol), social media and gaming are associated with more problematic use (40, 60, 61).

One other possibility is that individuals may use social media as a means to cope with feelings of loneliness, which may in turn, relate to PSMU. Loneliness is consistently linked with poorer mental health and long-term implications on mental well-being (62). In fact, higher loneliness is significantly associated with higher depressive and anxiety symptoms, as well as video gaming disorder and sleep problems in children and adolescents (62). Evidence also suggests loneliness experienced by university students may mediate the relationship between social skills, including social sensitivity, expressivity, and control, and both anxiety and depressive disorders (9). Longitudinal studies have shown an increase in loneliness levels since the pandemic (62). In addition, experiences of social isolation coincided with an increase in feelings of loneliness and stress, which impacted and/or worsened the mental health of young adults (63, 64). Despite the majority of students in this sample reporting living with their parents/family, the average loneliness score remained moderate. This is indicative of the importance of social connectedness among university students. Social relationships often mitigate the effects of stressors on psychological, emotional and behavioral health (65). In fact, reduced social connectedness is predictive of perceived social isolation (66). As a result of the pandemic restrictions, first-year students, in particular, were deprived of forming social connections with their peers. Indeed, emerging reports suggest first-year students were at a higher risk for mental health symptoms (67).

When further exploring loneliness in the context of coping with social media, we found the relationship between coping via social media and PSMU was especially strong for those with high levels of loneliness. Associations between loneliness and social media use during the pandemic have been widely reported, with some studies even showing a dose-response relationship between the two variables (68). These data support findings from an earlier study showing that younger individuals between the ages of 18-34 also experienced higher levels of loneliness and were more likely to use social media to cope with reduced social contact during the pandemic (69). Thus, our data provide compelling evidence that loneliness and coping with the stress of the pandemic is associated with increased PSMU among university students. In addition, these findings support evidence that individuals experiencing higher levels of loneliness are more likely to use social media more persistently and problematically (70). Together, these and our data point to a role for loneliness as a potential moderating factor influencing social media use, which may explain some of the variability around the development of problematic social media use. Given the increasing reliance of social media among youth and young adults, research exploring what predicts and moderates the development of problematic use is of significant public health concern, particularly as the prevalence and incidence of mental health disorders rise globally (71).

There are some limitations that are important to consider when interpreting results from this study. This is a cross-sectional study obtained during the COVID-19 pandemic; therefore, little is known about how the trends observed in this study compare to individual mental health symptoms before the pandemic. Longitudinal data are needed in order to assess changes in mood and social media use over time, as well as to establish the directionality of the relationship between social media use and mental health symptoms. For example, longitudinal studies have found evidence of a bidirectional relationship between social media use and loneliness, anxiety and depression (72, 73). Furthermore, 72.2% of the participants in the current study identified as women, thus, data from male participants must be interpreted with caution given lower sampling availability. It is also important to note that although a moderating effect of loneliness was found, the effect size was quite small, thus caution should be used when interpreting the importance of this moderated relation. Lastly, this study did not examine specific behaviors associated with social media: different patterns of social media use have been found to have varying effects on mental health symptoms, in which passive use such as scrolling and looking at other people’s content, is associated with higher anxiety and depressive symptoms (74). In contrast, active use such as chatting, sharing posts and interacting with others is associated with lower anxiety and depression symptoms, even after controlling for time spent on social media. Thus, it is important to understand how these patterns of use help disentangle the relationships between coping with social media, PSMU and mental health symptoms.

## 5 Conclusion

The current study provides further evidence into the existing relationship between maladaptive coping with social media and mental health symptoms among university students. Findings from this study also highlight the role of loneliness in exacerbating problematic social media use in individuals heavily coping with social media. Using these results, this study helps clarify some of the complex relationships among coping with social media, mental health symptoms and PSMU. The findings from this study can be used to raise awareness about the growing negative mental health outcomes associated with the high level of coping using social media. Future studies should further examine these relationships in longitudinal studies to help delineate the causal relationships among maladaptive coping, mental health symptoms and PSMU and investigate how different patterns of use of social media can serve as factors of risk or resilience to developing PSMU.

## Data availability statement

The datasets presented in this article are not readily available because these data were not approved to be shared outside of the research team. Requests to access the datasets should be directed to Kim G.C. Hellemans, kimhellemans@cunet.carleton.ca.

## Ethics statement

The studies involving humans were approved by Carleton University Research Ethics Board-B (CUREB-B). The studies were conducted in accordance with the local legislation and institutional requirements. The participants provided their written informed consent to participate in this study.

## Author contributions

KH, LK, RM and LG contributed to the conception and design of the study. LG performed the statistical analyses. LG, LK, and HS wrote first draft of the manuscript. LG, HS, LK, KH and RM each wrote sections of the manuscript. All authors contributed to the article and approved the submitted version.

## References

[1] Denovan A Macaskill A. An interpretative phenomenological analysis of stress and coping in first year undergraduates. Br Educ Res J (2013) 39(6):1002–24. doi: 10.1002/berj.3019

[2] Zaleski EH Levey-Thors C Schiaffino KM. Coping mechanisms, stress, social support, and health problems in college students. Appl Dev Sci (1998) 2(3):127–37. doi: 10.1207/s1532480xads0203_2

[3] Auerbach RP Mortier P Bruffaerts R Alonso J Benjet C Cuijpers P. WHO World Mental Health Surveys International College Student Project: Prevalence and distribution of mental disorders. J Abnorm Psychol (2018) 127(7):623–38. doi: 10.1037/abn0000362 PMC6193834 30211576

[4] American College Health Association. American College Health Association-National College Health Assessment III: Canadian Reference Group Executive Summary Spring 2022. Silver Spring, MD: American College Health Association (2022).

[5] Laustsen LM Christiansen J Maindal HT Plana-Ripoll O Lasgaard M. The longitudinal relation between loneliness and perceived stress: A structural equation modelling analysis of 10,159 individuals. Scand J Public Health (2023). doi: 10.1177/14034948231151716 36794680

[6] Erzen E Çikrikci Ö. The effect of loneliness on depression: A meta-analysis. Int J Soc Psychiatry (2018) 64(5):427–35. doi: 10.1177/0020764018776349 29792097

[7] Ge L Yap CW Ong R Heng BH. Social isolation, loneliness and their relationships with depressive symptoms: A population-based study. PloS One (2017) 12(8):e0182145. doi: 10.1371/journal.pone.0182145 28832594 PMC5568112

[8] McQuaid RJ Cox SML Ogunlana A Jaworska N. The burden of loneliness: Implications of the social determinants of health during COVID-19. Psychiatry Res (2021) 296:113648. doi: 10.1016/j.psychres.2020.113648 33348199 PMC9754822

[9] Moeller RW Seehuus M. Loneliness as a mediator for college students’ social skills and experiences of depression and anxiety. J Adolesc (2019) 73(1):1–13. doi: 10.1016/j.adolescence.2019.03.006 30933717 PMC6534439

[10] Santini ZI Fiori KL Feeney J Tyrovolas S Haro JM Koyanagi A. Social relationships, loneliness, and mental health among older men and women in Ireland: A prospective community-based study. J Affect Disord (2016) 204:59–69. doi: 10.1016/j.jad.2016.06.032 27337705

[11] Domènech-Abella J Mundó J Haro JM Rubio-Valera M. Anxiety, depression, loneliness and social network in the elderly: Longitudinal associations from The Irish Longitudinal Study on Ageing (TILDA). J Affect Disord (2019) 246:82–8. doi: 10.1016/j.jad.2018.12.043 30578950

[12] Eden AL Johnson BK Reinecke L Grady SM. Media for coping during COVID-19 social distancing: stress, anxiety, and psychological well-being. Front Psychol (2020) 11. doi: 10.3389/fpsyg.2020.577639 PMC7775316 33391094

[13] Kardefelt-Winther D. A conceptual and methodological critique of internet addiction research: Towards a model of compensatory internet use. Comput Hum Behav (2014) 31:351–4. doi: 10.1016/j.chb.2013.10.059

[14] Elhai JD Yang H Dempsey AE Montag C. Rumination and negative smartphone use expectancies are associated with greater levels of problematic smartphone use: A latent class analysis. Psychiatry Res (2020) 285:112845. doi: 10.1016/j.psychres.2020.112845 32045821

[15] Elhai JD Rozgonjuk D Yildirim C Alghraibeh AM Alafnan AA. Worry and anger are associated with latent classes of problematic smartphone use severity among college students. J Affect Disord (2019) 246:209–16. doi: 10.1016/j.jad.2018.12.047 30583147

[16] van Deursen AJAM Bolle CL Hegner SM Kommers PAM. Modeling habitual and addictive smartphone behavior. Comput Hum Behav (2015) 45:411–20. doi: 10.1016/j.chb.2014.12.039

[17] Franco JA Carrier LM. Social media use and depression, anxiety, and stress in Latinos: A correlational study. Hum Behav Emerg Technol (2020) 2(3):227–41. doi: 10.1002/hbe2.205

[18] Demirci K Akgönül M Akpinar A. Relationship of smartphone use severity with sleep quality, depression, and anxiety in university students. J Behav Addict (2015) 4(2):85–92. doi: 10.1556/2006.4.2015.010 26132913 PMC4500888

[19] Andreassen CS. Online social network site addiction: A comprehensive review. Curr Addict Rep (2015) 2(2):175–84. doi: 10.1007/s40429-015-0056-9

[20] Bányai F Zsila Á Király O Maraz A Elekes Z Griffiths MD. Problematic social media use: results from a large-scale nationally representative adolescent sample. PloS One (2017) 12(1):e0169839. doi: 10.1371/journal.pone.0169839 28068404 PMC5222338

[21] Cunningham S Hudson CC Harkness K. Social media and depression symptoms: a meta-analysis. Res Child Adolesc Psychopathol (2021) 49(2):241–53. doi: 10.1007/s10802-020-00715-7 33404948

[22] Primack BA Shensa A Escobar-Viera CG Barrett EL Sidani JE Colditz JB. Use of multiple social media platforms and symptoms of depression and anxiety: A nationally-representative study among U.S. Young Adults Comput Hum Behav (2017) 69:1–9. doi: 10.1016/j.chb.2016.11.013 PMC13268370 42305949

[23] Becker MW Alzahabi R Hopwood CJ. Media multitasking is associated with symptoms of depression and social anxiety. Cyberpsychol Behav Soc Netw (2013) 16(2):132–5. doi: 10.1089/cyber.2012.0291 23126438

[24] You Y Yang-Huang J Raat H Van Grieken A. Social media use and health-related quality of life among adolescents: cross-sectional study. JMIR Ment Health (2022) 9(10):e39710. doi: 10.2196/39710 36194460 PMC9579926

[25] Yoon S Kleinman M Mertz J Brannick M. Is social network site usage related to depression? A meta-analysis of Facebook–depression relations. J Affect Disord (2019) 248:65–72. doi: 10.1016/j.jad.2019.01.026 30711871

[26] Huang C. Time spent on social network sites and psychological well-being: A meta-analysis. Cyberpsychol Behav Soc Netw (2017) 20(6):346–54. doi: 10.1089/cyber.2016.0758 28622031

[27] Shannon H Bush K Villeneuve PJ Hellemans KG Guimond S. Problematic social media use in adolescents and young adults: systematic review and meta-analysis. JMIR Ment Health (2022) 9(4):e33450. doi: 10.2196/33450 35436240 PMC9052033

[28] Boursier V Gioia F Musetti A Schimmenti A. Facing loneliness and anxiety during the COVID-19 isolation: the role of excessive social media use in a sample of Italian adults. Front Psychiatry (2020) 11. doi: 10.3389/fpsyt.2020.586222 PMC7752864 33363484

[29] Ye Y Lin L. Examining relations between locus of control, loneliness, subjective well-being, and preference for online social interaction. Psychol Rep (2015) 116(1):164–75. doi: 10.2466/07.09.PR0.116k14w3 25621672

[30] Huang C. A meta-analysis of the problematic social media use and mental health. Int J Soc Psychiatry (2022) 68(1):12–33. doi: 10.1177/0020764020978434 33295241

[31] Taylor Z Yankouskaya A Panourgia C. Social media use, loneliness and psychological distress in emerging adults. Behav Inf Technol (2023) 1–14. doi: 10.1080/0144929X.2023.2209797

[32] Wickens CM McDonald AJ Elton-Marshall T Wells S Nigatu YT Jankowicz D. Loneliness in the COVID-19 pandemic: Associations with age, gender and their interaction. J Psychiatr Res (2021) 136:103–8. doi: 10.1016/j.jpsychires.2021.01.047 PMC8635289 33582608

[33] Bu F Steptoe A Fancourt D. Who is lonely in lockdown? Cross-cohort analyses of predictors of loneliness before and during the COVID-19 pandemic. Public Health (2020) 186:31–4. doi: 10.1016/j.puhe.2020.06.036 PMC7405905 32768621

[34] Geirdal AØ Ruffolo M Leung J Thygesen H Price D Bonsaksen T. Mental health, quality of life, wellbeing, loneliness and use of social media in a time of social distancing during the COVID-19 outbreak. A cross-country comparative study. J Ment Health (2021) 30(2):148–55. doi: 10.1080/09638237.2021.1875413 33689546

[35] Wenig V Heumann E Stock C Busse H Negash S Pischke CR. Associations of loneliness with mental health and with social and physical activity among university students in Germany: results of the COVID-19 German student well-being study (C19 GSWS). Front Public Health (2023) 11. doi: 10.3389/fpubh.2023.1284460 PMC10668152 38026349

[36] Prowse R Sherratt F Abizaid A Gabrys RL Hellemans KGC Patterson ZR. Coping with the COVID-19 pandemic: examining gender differences in stress and mental health among university students. Front Psychiatry (2021) 12. doi: 10.3389/fpsyt.2021.650759 PMC8058407 33897499

[37] Ellis WE Dumas TM Forbes LM. Physically isolated but socially connected: Psychological adjustment and stress among adolescents during the initial COVID-19 crisis. Can J Behav Sci (2020) 52(3):177–87. doi: 10.1037/cbs0000215

[38] Thompson RR Garfin DR Holman EA Silver RC. Distress, worry, and functioning following a global health crisis: A national study of Americans’ Responses to Ebola. Clin psychol Science (2017) 5(3):513–21. doi: 10.1177/2167702617692030

[39] Garfin DR Holman EA Silver RC. Cumulative exposure to prior collective trauma and acute stress responses to the boston marathon bombings. Psychol Sci (2015) 26(6):675–83. doi: 10.1177/0956797614561043 25896419

[40] Moretta T Buodo G Santucci VG Chen S Potenza MN. Problematic social media use is statistically predicted by using social media for coping motives and by positive reinforcement processes in individuals with high COVID-19-related stress levels. J Psychiatr Res (2023) 158:104–13. doi: 10.1016/j.jpsychires.2022.12.036 36580866

[41] Di Blasi M Salerno L Albano G Caci B Esposito G Salcuni S. A three-wave panel study on longitudinal relations between problematic social media use and psychological distress during the COVID-19 pandemic. Addictive Behav (2022) 134:107430. doi: 10.1016/j.addbeh.2022.107430 PMC9287460 35870439

[42] Casagrande M Favieri F Tambelli R Forte G. The enemy who sealed the world: effects quarantine due to the COVID-19 on sleep quality, anxiety, and psychological distress in the Italian population. Sleep Med (2020) 75:12–20. doi: 10.1016/j.sleep.2020.05.011 32853913 PMC7215153

[43] Gao J Zheng P Jia Y Chen H Mao Y Chen S. Mental health problems and social media exposure during COVID-19 outbreak. PloS One (2020) 15(4):e0231924. doi: 10.1371/journal.pone.0231924 32298385 PMC7162477

[44] Thomas AC Sullivan GB Allen FCL. A theoretical model of EGM problem gambling: more than a cognitive escape. Int J Ment Health Addict (2009) 7(1):97–107. doi: 10.1007/s11469-008-9152-6

[45] Cooper ML Russell M Skinner JB Frone MR Mudar P. Stress and alcohol use: Moderating effects of gender, coping, and alcohol expectancies. J Abnorm Psychol (1992) 101(1):139–52. doi: 10.1037/0021-843X.101.1.139 1537960

[46] Maroney N Williams BJ Thomas A Skues J Moulding R. A stress-coping model of problem online video game use. Int J Ment Health Addict (2019) 17(4):845–58. doi: 10.1007/s11469-018-9887-7

[47] Office of the Premier. Ontario Newsroom. Ontario Implementing Additional Public Health and Testing Measures to Keep People Safe. Toronto, Ontario: King's Printer for Ontario. (2020).

[48] Lovibond PF Lovibond SH. The structure of negative emotional states: Comparison of the Depression Anxiety Stress Scales (DASS) with the Beck Depression and Anxiety Inventories. Behav Res Ther (1995) 33(3):335–43. doi: 10.1016/0005-7967(94)00075-U 7726811

[49] Russell DW. UCLA loneliness scale (Version 3): reliability, validity, and factor structure. J Pers Assess (1996) 66(1):20–40. doi: 10.1207/s15327752jpa6601_2 8576833

[50] Andreassen CS Torsheim T Brunborg GS Pallesen S. Development of a facebook addiction scale. Psychol Rep (2012) 110(2):501–17. doi: 10.2466/02.09.18.PR0.110.2.501-517 22662404

[51] Hayes AF. Introduction to mediation, moderation, and conditional process analysis: a regression-based approach. 3rd ed. New York, NY: The Guilford Press (2022). Methodology in the social sciences.

[52] Pahayahay A Khalili-Mahani N. What media helps, what media hurts: A mixed methods survey study of coping with COVID-19 using the media repertoire framework and the appraisal theory of stress. J Med Internet Res (2020) 22(8):e20186. doi: 10.2196/20186 32701459 PMC7419155

[53] Woods R McInnis O Bedard M Asokumar A Santoni S Anisman H. Social support and unsupportive interactions in relation to depressive symptoms: Implication of gender and the BDNF polymorphism. Soc Neurosci (2020) 15(1):64–73. doi: 10.1080/17470919.2019.1650826 31364951

[54] Brammer WA Conn BM Iverson E Lankenau SE Dodson C Wong CF. Coping motives mediate the association of trauma history with problematic cannabis use in young adult medical cannabis patients and non-patient cannabis users. Subst Use Misuse (2022) 57(5):684–97. doi: 10.1080/10826084.2022.2026970 PMC11148629 35193442

[55] Thornton LK Baker AL Lewin TJ Kay-Lambkin FJ Kavanagh D Richmond R. Reasons for substance use among people with mental disorders. Addictive Behav (2012) 37(4):427–34. doi: 10.1016/j.addbeh.2011.11.039 22197045

[56] Patterson ZR Gabrys RL Prowse RK Abizaid AB Hellemans KGC McQuaid RJ. The influence of COVID-19 on stress, substance use, and mental health among postsecondary students. Emerg Adulthood (2021) 9(5):516–30. doi: 10.1177/21676968211014080

[57] Meng SQ Cheng JL Li YY Yang XQ Zheng JW Chang XW. Global prevalence of digital addiction in general population: A systematic review and meta-analysis. Clin Psychol Rev (2022) 92:102128. doi: 10.1016/j.cpr.2022.102128 35150965

[58] Violant-Holz V Gallego-Jiménez MG González-González CS Muñoz-Violant S Rodríguez MJ Sansano-Nadal O. Psychological health and physical activity levels during the COVID-19 pandemic: A systematic review. Int J Environ Res Public Health (2020) 17(24):9419. doi: 10.3390/ijerph17249419 33334073 PMC7765528

[59] Koob GF Volkow ND. Neurobiology of addiction: a neurocircuitry analysis. Lancet Psychiatry (2016) 3(8):760–73. doi: 10.1016/S2215-0366(16)00104-8 PMC6135092 27475769

[60] Lewis MA Hove MC Whiteside U Lee CM Kirkeby BS Oster-Aaland L. Fitting in and feeling fine: Conformity and coping motives as mediators of the relationship between social anxiety and problematic drinking. Psychol Addictive Behav (2008) 22(1):58–67. doi: 10.1037/0893-164X.22.1.58 18298231

[61] Melodia F Canale N Griffiths MD. The role of avoidance coping and escape motives in problematic online gaming: A systematic literature review. Int J Ment Health Addict (2022) 20(2):996–1022. doi: 10.1007/s11469-020-00422-w

[62] Farrell AH Vitoroulis I Eriksson M Vaillancourt T. Loneliness and well-being in children and adolescents during the COVID-19 pandemic: A systematic review. Children (2023) 10(2):279. doi: 10.3390/children10020279 36832408 PMC9955087

[63] Hwang TJ Rabheru K Peisah C Reichman W Ikeda M. Loneliness and social isolation during the COVID-19 pandemic. Int Psychogeriatr (2020) 32(10):1217–20. doi: 10.1017/S1041610220000988 PMC7306546 32450943

[64] Statistics Canada. Canadians’ Mental Health During the COVID-19 Pandemic (2020). Available at: https://www150.statcan.gc.ca/n1/en/daily-quotidien/200527/dq200527b-eng.pdf?st=v7r0OUez (Accessed cited 2023 Apr 29).

[65] Haslam C Cruwys T Haslam SA. “The we’s have it”: Evidence for the distinctive benefits of group engagement in enhancing cognitive health in aging. Soc Sci Med (2014) 120:57–66. doi: 10.1016/j.socscimed.2014.08.037 25222136

[66] Santini ZI Jose PE York Cornwell E Koyanagi A Nielsen L Hinrichsen C. Social disconnectedness, perceived isolation, and symptoms of depression and anxiety among older Americans (NSHAP): a longitudinal mediation analysis. Lancet Public Health (2020) 5(1):e62–70. doi: 10.1016/S2468-2667(19)30230-0 31910981

[67] Fruehwirth JC Biswas S Perreira KM. The Covid-19 pandemic and mental health of first-year college students: Examining the effect of Covid-19 stressors using longitudinal data. PloS One (2021) 16(3):e0247999. doi: 10.1371/journal.pone.0247999 33667243 PMC7935268

[68] Alheneidi H AlSumait L AlSumait D Smith AP. Loneliness and problematic internet use during COVID-19 lock-down. Behav Sci (2021) 11(1):5. doi: 10.3390/bs11010005 33418914 PMC7825032

[69] Lisitsa E Benjamin KS Chun SK Skalisky J Hammond LE Mezulis AH. Loneliness among young adults during covid-19 pandemic: the mediational roles of social media use and social support seeking. J Soc Clin Psychol (2020) 39(8):708–26. doi: 10.1521/jscp.2020.39.8.708

[70] O’Day EB Heimberg RG. Social media use, social anxiety, and loneliness: A systematic review. Comput Hum Behav Rep (2021) 3:100070. doi: 10.1016/j.chbr.2021.100070

[71] Racine N McArthur BA Cooke JE Eirich R Zhu J Madigan S. Global prevalence of depressive and anxiety symptoms in children and adolescents during COVID-19. JAMA Pediatr (2021) 175(11):1142. doi: 10.1001/jamapediatrics.2021.2482 34369987 PMC8353576

[72] Zhou W Yan Z Yang Z Hussain Z. Problematic social media use and mental health risks among first-year Chinese undergraduates: a three-wave longitudinal study. Front Psychiatry (2023) 14. doi: 10.3389/fpsyt.2023.1237924 PMC10512716 37743982

[73] Li JB Mo PKH Lau JTF Su XF Zhang X Wu AMS. Online social networking addiction and depression: The results from a large-scale prospective cohort study in Chinese adolescents. J Behav Addict (2018) 7(3):686–96. doi: 10.1556/2006.7.2018.69 PMC6426399 30203664

[74] Thorisdottir IE Sigurvinsdottir R Asgeirsdottir BB Allegrante JP Sigfusdottir ID. Active and passive social media use and symptoms of anxiety and depressed mood among Icelandic adolescents. Cyberpsychol Behav Soc Netw (2019) 22(8):535–42. doi: 10.1089/cyber.2019.0079 31361508

